# Verification of systems biology research in the age of collaborative competition

**DOI:** 10.1186/1753-6561-6-S6-P48

**Published:** 2012-10-01

**Authors:** Carine Poussin

**Affiliations:** 1 Pathway Informatics, Philip Morris International, Research & Development, Neuchâtel, Switzerland

## 

Modern society demands greater scrutiny of the potential health risks and benefits of long-term, and sometimes lifelong, exposure to drugs, chemicals, and substances found in consumer products and the environment. Organizations such as companies and academic consortia conduct large multi-year scientific studies that entail the collection and analysis of thousands of data points. The individual experiments are often conducted over many physical sites and with internal and outsourced components. To extract maximum value, the interested parties need to verify the accuracy and reproducibility of automated collection and analysis workflows in systems biology before the initiation of large multi-year studies.

Traditional verification using the peer-review process has shortcomings, such as lack of scalability, which renders it insufficient for the assessment of high-throughout research. A team of researchers at Philip Morris International (PMI) and IBM, whose aim is to improve the effectiveness of scientific studies and the verification of scientific findings, propose a scheme called IMPROVER, for Industrial Methodology for Process Verification of Research. This methodology evaluates a research program by dividing its workflow into smaller building blocks, whereby the verification of each building block can be done internally or externally via challenge-based 'crowd-sourcing' to a research community.

Scientific challenges will be broadcast to potential stakeholders in the form of an open call for participation, with the intention of providing the community with the opportunity to test their computational methods on new data as well as to partake in a collaborative effort whose ultimate goal could contribute to solving a grand scientific problem.

Considering cancer as the leading cause of death worldwide, we formulate the Diagnostics Signature Challenge to evaluate novel approaches for the identification of robust and predictive signatures for this disease. The goal of a Diagnostics Signature Challenge is to verify that transcriptomics data contain enough information for the determination and prognosis of certain human disease states that could profit from better diagnostics signatures. Here we will describe the approach, the necessary operational steps, and how we intend to engage the wider scientific community to assess the applicability of the IMPROVER approach to molecular diagnostics (that is, genomic signatures).

